# Metacarpal lengthening by distraction histiogenesis in adults

**DOI:** 10.4103/0019-5413.55467

**Published:** 2009

**Authors:** Sakti Prasad Das, Ram Narayan Mohanty, Sanjay Kumar Das

**Affiliations:** Swami Vivekanand National Institute of Rehabilitation Training and Research, Olatpur, Bairoi, Cuttack, Orissa, India

**Keywords:** Metacarpal lengthening, thumb lengthening, distraction histeogenesis

## Abstract

**Background:**

Metacarpal lengthening in the hand is a new application for distraction neo-histiogenesis. Metacarpal lengthening with distraction helps in improvement in pinch function. Thumb lengthening is technically easy in comparison to other metacarpals. We present the operative treatment and post-operative outcome in nine patients with amputations and congenital anomalies.

**Materials and Methods:**

Nine patients underwent distraction osteogenesis for the treatment of amputations of the hand and other congenital anomalies. The dominant right hand was operated in eight cases and the left hand in one case. There were six males and three females. Improvement of function was always the aim of surgery. Age range was between 18 and 23 years. Thumb lengthening was performed in five patients and that of the index finger in four patients. Distraction started on the fifth post-operative day at the rate of 0.25 mm/day. Sensory function and bone consolidation was assessed before fixator removal.

**Results:**

The mean duration of distraction was 51 days (range, 42–60 days) and the distractor was removed at a mean of 150 days (range, 140 and 160 days) and the bones were lengthened by a mean of 24 mm (range, 20–28 mm) There was improvement of function in all cases.

**Conclusion:**

The metacarpal lengthening by distraction histiogenesis in congenital and traumatic amputations is safe and simple method to improve pinch function of hand.

## INTRODUCTION

Pioneered in Russia in the 1950s, the Ilizarov technique is used for the correction of various types of deformities and also for restoration of limb length. The use of distraction histiogenesis in metacarpals and metatarsals is a new concept.^1^–^9^ These techniques have had some inherent problems like pin tract infection, non-union, and metacarpo phalangeal joint stiffness. But, the lengthened fingers help to improve functions and position of hand in space. Metacarpal lengthening in congenital amputations helps to improve pinch and grip.

There are so many other highly skilled techniques like microvascular toe or tissue transplantation and pollicization that are possible,^10^ but these are confined to selected centers only. Distraction lengthening is within the reach of all orthopedic surgeons. Now a days, miniaturized stable external fixators are available that can be applied for the above-said procedures.

We have performed metacarpal lengthening by distraction histiogenesis with small external fixator in nine hands. We describe the operative procedures and our results.

## MATERIALS AND METHODS

From 2001 to 2006, we treated nine hands in nine patients by distraction neo-histiogenesis. In five patients, the cause was traumatic amputation and in four patients it was congenital anomalies. Of the four, two had absence of fingers from the level of base of proximal phalanx and two were associated with syndactylism. There was no neurovascular deficit in any of the cases and the available muscle functions were normal. Length intended for lengthening was around 2 cm in all cases. Objective of lengthening was functional improvement. There were six males and three females. The right hand was involved in all cases but one. Only thumb metacarpal lengthening was performed in five patients and index finger lengthening was performed in four patients. Traumatic cases, thumb metacarpal lengthening was performed in three and index metacarpal in two cases. Age range was between 18 and 23 years. Clinical details of the patients are given in Table 1.

**Table 1:** Clinical details of the patients

Age (years)/sex	Side	Level of amputation	Traumatic/congenital	Lengthening achieved (in mm)	Consolidation (in days)	Distractor removed (in days)	K-wire removed (in days)	Secondary procedure	Hand function	Complications
18/M	R	Base of proximal phalanx	Traumatic	20	85	153	175	Nil	Pinch	Nil
19/M	R	-Do-	Traumatic	22	75	142	162	Nil	Pinch and grasp	Hyperextension deformity
22/M	R	MP Joint	Congenital	20	70	140	149	Bone grafting	Pinch	Non-union, pin loosening
23/M	R	Base of proximal phalanx	Congenital	28	98	152	163	Bone grafting	Pinch and grasp	Non-union
19/F	L	-Do-	Traumatic	23	110	160	167	Nil	-Do-	Hyperextension deformity
21/M	R	-Do-	Congenital	26	106	158	162	Nil	-Do-	-Do-
20/F	R	MP joint	Traumatic	28	86	145	163	Nil	-Do-	Nil
19/F	R	MP joint	Traumatic	22	89	148	150	Nil	-Do-	Nil
20/M	R	MP joint	Congenital	24	90	156	166	Nil	Pinch	Hyperextension deformity

The planned rate of lengthening was 0.25 mm/day. The post-operative rate of bone lengthening was analyzed on the basis of anteroposterior radiographs made at the time of distractor removal. Regenerate, pinch function, and sensory function in the treated hand were evaluated.

### Operative procedure

With the patient under brachial anesthesia, a straight incision was made on the dorsum of the first or second ray of hand and the metacarpal was exposed. The periosteum was dissected longitudinally and osteotomy was made horizontally toward the metaphysial area of the bone. A 1 mm K-wire was placed in the metacarpal to maintain alignment. The periosteum and the skin were sutured in layers. Two 2 mm K-wires were inserted 2 mm proximal and distal to the osteotomy site. The distractor device was then assembled to pins [Figures 1 and 2]. Distraction was started 5 days post-operatively at a planned rate of 0.25 mm/day and was continued by the patient himself. X-ray was taken at every week till 3 weeks then every 3 weeks during the distraction and consolidation periods. The distraction device was removed once the desired length was achieved and there was consolidation of at least three cortices. All cases were followed-up from 2 to 4 years.

**Figure 1A:**
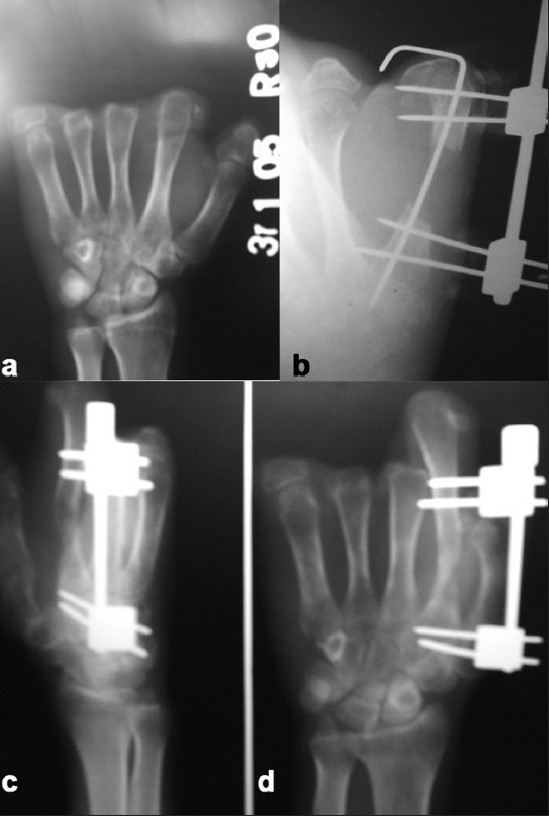
(a) X-ray (anteroposterior view) of hand showing amputated phalanges. (b) X-ray (anteroposterior view) of hand showing metacarpal lengthening in progress; K-wire and distractor *in situ*. (c, d) X-ray (oblique and anteroposterior views) of hand showing regenerate with distractor in situ

**Figure 1B:**
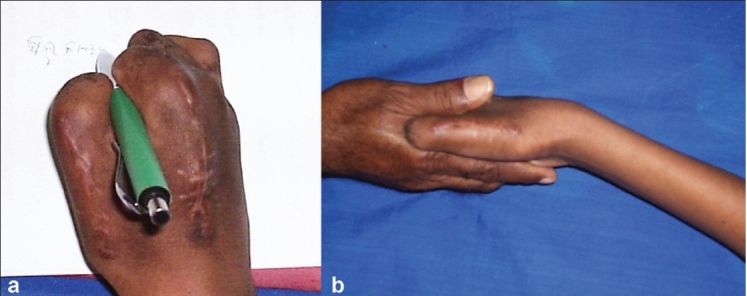
Clinical photographs of hand (a,b) showing hand functions

**Figure 2:**
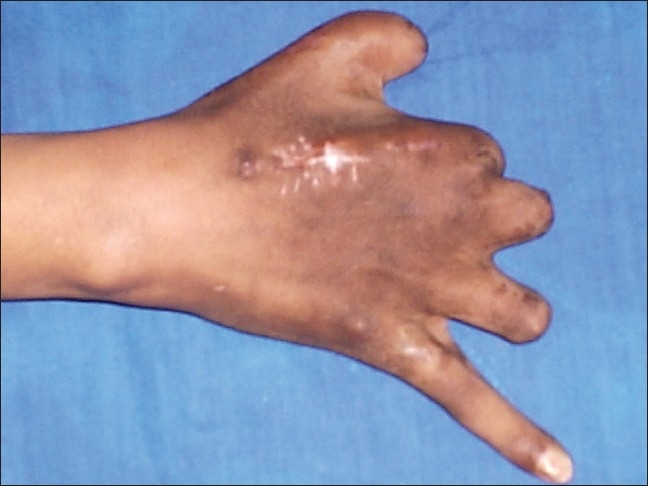
Clinical photograph of hand showing surgical scar mark and lengthened second ray

## RESULTS

The mean duration of the distraction is 51 days (42–60 days) and of the consolidation is 90 days (70–110 days). The distraction device was removed at a mean of 150 days (140–160 days). The bones were lengthened by a mean of 24 mm (20–28 mm). On an average, a 20% increase in length was achieved. In one case there was pin loosening and in two cases non-union led to angular deformity. Hyperextension deformity of the metacarpophalangeal joint is a common complication that occurred in four of nine cases. Six months after operation, all hand movements were normal except fully active flexion of the lengthened finger. There was intact sensation of the lengthened rays. There was non-union in two cases, which were then grafted and union was achieved.

## DISCUSSION

The principle of distraction osteogenesis can be very effectively and rewardingly applied to the small bones of the hand. It can provide a source for enhanced prehension and gain functional length.^1^ This was used in this study in cases of traumatic amputation of phalanges and congenital amputation through phalanges. In this study, the index finger and thumb were lengthened. Length of the finger is important for prehension, stability, and positioning in space. In our cases, the metacarpals were available to achieve the above objectives. Distraction lengthening does not replace toe transplantation or policization. Rather, it adds another tool for these challenging problems. Toe transplantation is out of reach in most centers. Till today, we have not yet been able to develop a functional hand or finger prosthesis for various amputations of the hand at the phalangeal or metacarpal level.

Metacarpals were selected for lengthening due to their good vascular supply and adequate soft tissue coverage. Presence of cancellous bone and stability of the base of the metacarpal determines the level of osteotomy between base and shaft. Previous methods of lengthening by osteotomy, iliac crest grafting, and immobilization by POP cast leads to stiffness of the metacarpophalangeal joint. Distraction lengthening allows activity of the hand during lengthening as well as physiotherapy to the metacarpophalangeal joint to prevent stiffness. Bone grafting is not needed in these cases.

There are few surgical options for the reconstruction of pinch function in patients with amputations of the thumb. The thumb is so small that opposition is often severely limited. Metacarpal lengthening in the thumb was adequate to allow for pinch function. The thumb metacarpal is an independent bone in comparison with other metacarpals. Hence, in our experience, thumb lengthening is easy in comparison with others.

The rate of lengthening was 0.25 mm/day. On the fifth post-operative day, change of dressing was done and family members were taught pin site care as well as method of distraction. Pin site care includes once daily cleaning with hydrogen peroxide. Swabs and betadine-soaked dressings were applied. 0.25 mm distraction daily is painless and well tolerated. Patients were encouraged to use their hand during the treatment period.

Consolidation was evaluated by taking antero posterior and lateral X-rays. We usually check for the formation of a minimum of three complete cortices before removal of the distractor.

Pin loosening, nonunion, and metacarpophalangeal joint stiffness are some of the complications encountered. Mobilization of the metacarpophalangeal joint and active use of the hand were encouraged.

Improvement of pinch and grasp functions were observed. Patients were able to write and button and unbutton dresses. Patients were operated for functional improvement. Cosmesis was never a criterion.

Functional improvement by toe transfer is excellent. But, secondary operations like tenolysis, repair of ruptured tendon, tendon grafting or transfers, opponensplasty, and ligamentoplasty for joint stability were necessary in all cases^10^. Toe transfer is a highly skilled and costly treatment for our poor patients. They need only gross functional improvement in low cost.

Technique-wise, use of K-wire advocated by Takeshi Miyawalki *et al*.,^6^ which prevents angulations and maintains alignments as compared with I.A Mansur^8^ and Seitz W H Jr.^3^ I A Mansur advocated osteotomy, spreading, and iliac crest grafting, which were not followed in our cases.

In children, the rate of distraction was 1 mm/day^3^ and 0.6 mm/day.^6^ In adults, callus formation is slow; hence we distract at rate of 0.25mm/d and we are satisfied with our results. Matev^5^ recommended a lengthening rate of 1.5 mm/day and Takeshi Miyawaki *et al*.^6^ recommended the rate to be 0.6 mm/day. We found that the rate of lengthening of 0.25 mm/day is suitable to us. The device is easily operated to the desired length and can be removed on an outpatient basis. Bone distraction requires a longer treatment period. But, the lengthened rays have an intact sensation. Counseling is very important in these treatments because patient cooperation is essential.

Inadequate bone formation, angular deformity, metacarpophalangeal joint stiffness, pin tract infection, and loosening are associated with this technique and care should be taken to avoid these complications.^2^–^4^

It has been seen that inadequate bone formation is more common in the second metacarpal than the in thumb. The quality of regeneration was better in the thumb than in the second metacarpal, as seen radiologically, with no previous reports. This is probably dependant on the surrounding soft tissues. The thumb metacarpal is surrounded by muscles all around with a rich blood supply than the index finger. Careful handling of the periosteum and osteotomy in the metaphysical area prevents such complications. In this series, two cases (both in the second metacarpal) had poor bone formation leading to non-union and required secondary procedures in the form of antologous cancellous bone grafting. Pin tract loosening, which is a common complication, can be minimized by meticulous post-operative pin site care. We had one case of pintract loosening in index finger lengthening change pin to a more distal site (single pin only).

Pensler *et al*.^7^ reported that 17% of the digits had angular deformity that necessitated reoperation. We also faced the same problem in the first two cases and that complication has been prevented with the use of intramedullary K-wires, which are removed after consolidation of the bone regenerate. As hyperextension deformity is very common, we try to keep the intramedullary K-wires dorsal to the metacarpophalangeal joint.

## CONCLUSION

Metacarpal lengthening was found to be a simple, safe, and cost-effective method of reconstruction that provides a permanent improvement in pinch function. Holding small objects, writing, and buttoning and unbuttoning of shirts were some of the improvements in hand function post-operatively in comparison with the absence of the same functions pre-operatively.
